# Supplementary material for: When do science recommendations stop being effective? The case of the sprawl of diesel electricity generators in Beirut
Source: PLoS One. 2024 Dec 31;19(12):e0313341. doi: 10.1371/journal.pone.0313341 (PMC11687642; doi:10.1371/journal.pone.0313341)
Supplement: S1 File — (DOCX) [file pone.0313341.s001.docx]

**When do science recommendations stop being effective? The Case of the sprawl of diesel electricity generators in Beirut**

Mohammad Al Hallak^1^, Abdul Aziz Kurdieh^1,#a^, Amira Yassine^1,#b^,Rachel El Hage^1^ and Najat Saliba^1^*

^1^ Department of Chemistry, American University of Beirut, Beirut, Lebanon

^#a^Laboratory of Atmospheric Chemistry, Paul Scherrer Institut (PSI), Villigen 5232, Switzerland

^#b^ Environmental Health and Engineering Department, Johns Hopkins University, Baltimore, USA

*Corresponding Author

*Corresponding Author:

Email: [najataoun.mp@gmail.com](mailto:najataoun.mp@gmail.com) and [ns30@aub.edu.lb](mailto:ns30@aub.edu.lb) (NS)

**Supporting Information**

**Results**

**S1 Text. Emissions of PM_2.5_  at the three sites in Beirut**

S1A Table. PM_2.5_ levels, in μg/m^3^, at Beirut Central District (BCD) for every sampling point

|  | **Date** | **PM_2.5_ level in μg/m³** |
| --- | --- | --- |
| **Beirut Central District (BCD)** | 3/1/2022 | 16.24 |
|  | 3/8/2022 | 11.81 |
|  | 3/14/2022 | 11.40 |
|  | 3/20/2022 | 26.66 |
|  | 3/26/2022 | 14.65 |
|  | 4/1/2022 | 32.13 |
|  | 4/7/2022 | 27.57 |
|  | 4/13/2022 | 11.89 |
|  | 4/19/2022 | 12.29 |
|  | 4/25/2022 | 24.33 |
|  | 5/18/2022 | 23.91 |
|  | 10/5/2022 | 15.49 |
|  | 10/11/2022 | 17.22 |
|  | 10/18/2022 | 12.62 |
|  | 10/31/2022 | 16.75 |
|  | ***Average*** | 18.33 |
|  | ***Standard deviation*** | 6.78 |
|  | ***maximum*** | 32.13 |
|  | ***minimum*** | 11.40 |

S1B Table. PM_2.5_ levels, in μg/m^3^, at American University of Beirut (AUB) for every sampling point

|  | **Date** | **PM_2.5_ level in μg/m³** |
| --- | --- | --- |
| **American University of Beirut (AUB)** | 6/1/2022 | 5.83 |
|  | 6/7/2022 | 18.95 |
|  | 6/19/2022 | 10.21 |
|  | 6/22/2022 | 16.15 |
|  | 7/7/2022 | 19.75 |
|  | 7/14/2022 | 15.41 |
|  | 7/20/2022 | 10.88 |
|  | 7/26/2022 | 9.69 |
|  | 8/1/2022 | 19.41 |
|  | 8/10/2023 | 10.35 |
|  | 8/24/2022 | 10.65 |
|  | 8/29/2022 | 14.74 |
|  | 9/13/2022 | 12.58 |
|  | 9/22/2022 | 9.49 |
|  | 11/29/2022 | 19.45 |
|  | 12/10/2022 | 21.1 |
|  | 12/16/2022 | 23.3 |
|  | 12/23/2022 | 15.1 |
|  | 12/28/2022 | 19.5 |
|  | 1/3/2023 | 14.41 |
|  | 1/9/2023 | 13.47 |
|  | 1/15/2023 | 5.33 |
|  | 1/22/2023 | 25.27 |
|  | 1/31/2022 | 7.08 |
|  | 2/2/2023 | 2.41 |
|  | 2/15/2023 | 10.33 |
|  | 2/21/2023 | 12.91 |
|  | 2/27/2023 | 25.66 |
|  | ***Average*** | 14.27 |
|  | ***Standard Deviation*** | 6.03 |
|  | ***Maximum*** | 25.66 |
|  | ***Minimum*** | 2.41 |

S1C Table. PM_2.5_ levels, in μg/m^3^, at Nursing School Makassed University (NSMU) for every sampling point

|  | **Date** | **PM_2.5_ level in μg/m³** |
| --- | --- | --- |
| **Nursing School Makassed University (NSMU)** | 4/11/2023 | 24.62 |
|  | 4/18/2023 | 60.32 |
|  | 5/6/2023 | 11.11 |
|  | 5/11/2023 | 29.44 |
|  | 5/18/2023 | 8.33 |
|  | 5/23/2023 | 15.74 |
|  | 5/31/2023 | 16.04 |
|  | 6/6/2023 | 31.01 |
|  | 6/21/2023 | 13.88 |
|  | 6/26/2023 | 18.05 |
|  | 7/6/2023 | 21.75 |
|  | 7/11/2023 | 20.83 |
|  | ***Average*** | 22.59 |
|  | ***Standard Deviation*** | 13.72 |
|  | ***Maximum*** | 60.32 |
|  | ***Minimum*** | 8.33 |

**S2 Text. The concentration of particle bound polycyclic aromatic hydrocarbons (PPAHs) at the three sites in Beirut**

S2A1 Table. Particle bound Polycyclic Aromatic Hydrocarbons (PPAHs) levels, in ng/m^3^, at Beirut Central District (BCD) for every sampling point (first part)

| Polycyclic Aromatic Hydrocarbons | Abbreviation | 3/9/2022 | 3/15/2022 | 3/21/2022 | 3/27/2022 | 4/2/2022 | 4/8/2022 | 4/14/2022 | 4/20/2022 |
| --- | --- | --- | --- | --- | --- | --- | --- | --- | --- |
| Naphtalene | Nap | 0.70 | 0.76 | 0.58 | 0.77 | 1.18 | 0.82 | 0.64 | 0.55 |
| Acenaphtylene | Acy | 0.32 | 0.37 | 0.45 | 0.33 | 0.55 | 0.39 | 0.34 | 0.23 |
| Acenaphtene | Ace | 0.45 | 0.62 | 0.47 | 0.20 | 0.38 | 0.42 | 0.42 | 0.49 |
| Fluorene | Flu | 0.42 | 0.47 | 0.43 | 0.54 | 0.82 | 0.55 | 0.61 | 0.45 |
| Phenanthrene | Phe | 0.57 | 1.23 | 1.80 | 0.81 | 1.57 | 0.86 | 0.87 | 0.50 |
| Anthracene | Ant | 0.67 | 0.94 | 1.47 | 0.73 | 1.01 | 0.78 | 0.70 | 0.54 |
| Fluoranthene | Flt | 1.14 | 2.46 | 4.65 | 1.63 | 2.96 | 1.97 | 1.49 | 0.45 |
| Pyrene | Pyr | 1.86 | 3.30 | 5.02 | 1.93 | 4.25 | 3.47 | 1.75 | 0.70 |
| benzo[a]anthracene | BaA | 1.28 | 2.86 | 3.47 | 1.96 | 4.50 | 2.85 | 0.88 | 0.73 |
| Chrysene | Chr | 2.05 | 4.74 | 6.29 | 2.86 | 6.14 | 4.49 | 1.82 | 1.20 |
| Benzo[k]fluoranthene | BkF | 1.94 | 4.24 | 6.09 | 2.47 | 9.90 | 3.83 | 1.67 | 1.22 |
| Benzo[b]fluoranthene | BbF | 0.99 | 2.10 | 3.02 | 1.37 | 4.14 | 1.80 | 0.86 | 0.70 |
| Benzo[a]pyrene | BaP | 1.56 | 2.85 | 3.93 | 1.68 | 8.59 | 2.95 | 0.90 | 0.82 |
| Benzo[g,h,i]perylene | BghiP | 0.05 | 0.07 | 0.05 | 0.17 | 0.21 | 0.47 | 0.08 | 0.17 |
| Dibenz[a,h]anthracene | DahA | 0.25 | 0.27 | 0.24 | 0.12 | 0.38 | 0.29 | 0.23 | 0.12 |
| Indeno[1,2,3-cd]pyrene | IP | 0.12 | 0.13 | 0.32 | 0.03 | 0.27 | 0.42 | 0.09 | 0.03 |
|  | Total | 14.36 | 27.43 | 38.29 | 17.60 | 46.84 | 26.36 | 13.35 | 8.92 |

S2A2 Table. Particle bound Polycyclic Aromatic Hydrocarbons (PPAHs) levels, in ng/m^3^, at Beirut Central District (BCD) for every sampling point (second part)

| Polycyclic Aromatic Hydrocarbons | Abbreviation | 4/26/2022 | 5/19/2022 | 10/12/2022 | 10/19/2022 | 11/2/2022 | Average in ng/m3 | Standard Deviation in ng/m3 |
| --- | --- | --- | --- | --- | --- | --- | --- | --- |
| Naphtalene | Nap | 0.66 | 0.78 | 0.92 | 0.60 | 0.75 | 0.75 | 0.17 |
| Acenaphtylene | Acy | 0.33 | 0.33 | 0.40 | 0.49 | 0.38 | 0.38 | 0.08 |
| Acenaphtene | Ace | 0.96 | 0.25 | 0.28 | 0.14 | 0.44 | 0.42 | 0.21 |
| Fluorene | Flu | 0.54 | 0.40 | 0.41 | 1.62 | 0.55 | 0.60 | 0.33 |
| Phenanthrene | Phe | 0.77 | 0.54 | 0.71 | 0.39 | 0.90 | 0.89 | 0.42 |
| Anthracene | Ant | 0.70 | 0.63 | 0.78 | 0.22 | 0.76 | 0.76 | 0.28 |
| Fluoranthene | Flt | 0.90 | 0.64 | 1.07 | 0.40 | 1.29 | 1.62 | 1.18 |
| Pyrene | Pyr | 1.32 | 0.92 | 1.56 | 0.82 | 1.76 | 2.20 | 1.37 |
| benzo[a]anthracene | BaA | 1.33 | 1.62 | 1.60 | 1.10 | 1.52 | 1.98 | 1.12 |
| Chrysene | Chr | 2.35 | 2.53 | 2.57 | 1.51 | 2.54 | 3.16 | 1.69 |
| Benzo[k]fluoranthene | BkF | 1.91 | 2.66 | 2.34 | 1.00 | 3.01 | 3.25 | 2.43 |
| Benzo[b]fluoranthene | BbF | 1.03 | 1.33 | 1.10 | 0.47 | 1.47 | 1.57 | 1.02 |
| Benzo[a]pyrene | BaP | 1.49 | 1.63 | 1.64 | 0.95 | 2.31 | 2.41 | 2.07 |
| Benzo[g,h,i]perylene | BghiP | 0.04 | 0.02 | 0.01 | 0.31 | 0.08 | 0.13 | 0.13 |
| Dibenz[a,h]anthracene | DahA | 0.22 | 0.36 | 0.27 | 0.04 | 0.25 | 0.24 | 0.09 |
| Indeno[1,2,3-cd]pyrene | IP | 0.09 | 0.20 | 0.14 | 0.15 | 0.09 | 0.16 | 0.11 |
|  | Total | 14.63 | 14.84 | 15.81 | 10.20 | 18.11 | 20.52 | 11.27 |

S2B1 Table. Particle bound Polycyclic Aromatic Hydrocarbons (PPAHs) levels, in ng/m^3^, at American University of Beirut (AUB) for every sampling point (first part)

| Polycyclic Aromatic Hydrocarbons | Abbreviation | 6/2/2022 | 6/8/2022 | 9/13/2022 | 9/22/2022 | 11/29/2022 | 12/11/2022 |
| --- | --- | --- | --- | --- | --- | --- | --- |
| Naphtalene | Nap | 0.64 | 0.73 | 0.67 | 0.76 | 0.89 | 0.74 |
| Acenaphtylene | Acy | 0.29 | 0.24 | 0.38 | 0.48 | 0.39 | 0.37 |
| Acenaphtene | Ace | 0.40 | 0.49 | 0.17 | 1.78 | 0.31 | 0.57 |
| Fluorene | Flu | 0.42 | 0.42 | 0.45 | 0.44 | 0.46 | 0.44 |
| Phenanthrene | Phe | 0.36 | 0.47 | 0.73 | 0.88 | 1.01 | 1.08 |
| Anthracene | Ant | 0.55 | 0.63 | 0.78 | 0.94 | 0.94 | 0.85 |
| Fluoranthene | Flt | 0.18 | 0.54 | 1.31 | 1.73 | 2.10 | 2.11 |
| Pyrene | Pyr | 0.19 | 0.69 | 1.55 | 2.13 | 3.02 | 2.43 |
| benzo[a]anthracene | BaA | 0.18 | 0.37 | 1.11 | 1.75 | 3.21 | 1.79 |
| Chrysene | Chr | 0.22 | 0.71 | 2.20 | 3.84 | 5.66 | 3.67 |
| Benzo[k]fluoranthene | BkF | 0.36 | 0.59 | 5.57 | 6.79 | 8.12 | 4.95 |
| Benzo[b]fluoranthene | BbF | 0.35 | 0.45 | 2.59 | 3.12 | 3.86 | 2.43 |
| Benzo[a]pyrene | BaP | 0.31 | 0.46 | 3.33 | 4.17 | 5.80 | 3.10 |
| Benzo[g,h,i]perylene | BghiP | 0.11 | 0.08 | 0.03 | 0.11 | 0.18 | 0.09 |
| Dibenz[a,h]anthracene | DahA | 0.16 | 0.24 | 0.24 | 0.25 | 0.21 | 0.21 |
| Indeno[1,2,3-cd]pyrene | IP | 0.09 | 0.11 | 0.10 | 0.34 | 0.22 | 0.27 |
|  | Total | 4.83 | 7.23 | 21.20 | 29.49 | 36.38 | 25.10 |

S2B2 Table. Particle bound Polycyclic Aromatic Hydrocarbons (PPAHs) levels, in ng/m^3^, at American University of Beirut (AUB) for every sampling point (second part)

| Polycyclic Aromatic Hydrocarbons | Abbreviation | 12/17/2022 | 12/29/2022 | 1/4/2023 | 1/10/2023 | 1/16/2023 | 1/23/2023 |
| --- | --- | --- | --- | --- | --- | --- | --- |
| Naphtalene | Nap | 0.90 | 0.92 | 0.90 | 0.60 | 0.83 | 0.83 |
| Acenaphtylene | Acy | 0.43 | 0.38 | 0.43 | 0.38 | 0.39 | 0.45 |
| Acenaphtene | Ace | 0.31 | 0.31 | 0.25 | 0.28 | 1.86 | 0.22 |
| Fluorene | Flu | 0.42 | 0.44 | 0.40 | 0.45 | 0.44 | 0.43 |
| Phenanthrene | Phe | 0.71 | 0.63 | 0.80 | 0.83 | 1.00 | 0.93 |
| Anthracene | Ant | 0.79 | 0.72 | 0.91 | 0.77 | 0.94 | 0.93 |
| Fluoranthene | Flt | 0.78 | 0.61 | 1.41 | 1.98 | 2.42 | 2.12 |
| Pyrene | Pyr | 1.27 | 0.94 | 2.13 | 2.14 | 4.05 | 2.82 |
| benzo[a]anthracene | BaA | 0.55 | 0.78 | 2.00 | 1.24 | 3.10 | 2.62 |
| Chrysene | Chr | 0.84 | 1.37 | 3.37 | 2.62 | 5.25 | 5.23 |
| Benzo[k]fluoranthene | BkF | 0.65 | 1.02 | 4.75 | 3.06 | 5.06 | 7.25 |
| Benzo[b]fluoranthene | BbF | 0.47 | 0.66 | 2.15 | 1.42 | 2.52 | 3.31 |
| Benzo[a]pyrene | BaP | 0.56 | 0.85 | 3.53 | 1.79 | 3.84 | 4.88 |
| Benzo[g,h,i]perylene | BghiP | 0.01 | 0.10 | 0.13 | 0.13 | 0.02 | 0.03 |
| Dibenz[a,h]anthracene | DahA | 0.20 | 0.20 | 0.22 | 0.18 | 0.17 | 0.26 |
| Indeno[1,2,3-cd]pyrene | IP | 0.13 | 0.08 | 0.13 | 0.08 | 0.12 | 0.31 |
|  | Total | 9.04 | 9.99 | 23.53 | 17.96 | 32.00 | 32.61 |

S2B3 Table. Particle bound Polycyclic Aromatic Hydrocarbons (PPAHs) levels, in ng/m^3^, at American University of Beirut (AUB) for every sampling point (third part)

| Abbreviation | 2/2/2023 | 2/3/2023 | 2/16/2023 | 2/22/2023 | 2/28/2023 | Average in ng/m3 | Standard Deviation in ng/m3 |
| --- | --- | --- | --- | --- | --- | --- | --- |
| Nap | 0.75 | 0.57 | 0.60 | 0.68 | 0.66 | 0.75 | 0.12 |
| Acy | 0.33 | 0.29 | 0.26 | 0.41 | 0.35 | 0.37 | 0.07 |
| Ace | 0.18 | 1.54 | 0.19 | 0.23 | 0.32 | 0.55 | 0.57 |
| Flu | 0.42 | 0.40 | 0.46 | 0.41 | 0.41 | 0.43 | 0.02 |
| Phe | 0.60 | 0.52 | 0.83 | 0.52 | 0.51 | 0.73 | 0.21 |
| Ant | 0.78 | 0.63 | 0.79 | 0.68 | 0.66 | 0.78 | 0.12 |
| Flt | 1.22 | 0.78 | 1.53 | 0.86 | 0.76 | 1.32 | 0.67 |
| Pyr | 1.66 | 1.15 | 1.17 | 0.97 | 0.76 | 1.71 | 0.99 |
| BaA | 1.67 | 1.20 | 0.37 | 0.73 | 0.36 | 1.35 | 0.96 |
| Chr | 2.56 | 1.80 | 0.94 | 1.56 | 0.74 | 2.50 | 1.74 |
| BkF | 2.30 | 1.58 | 1.28 | 2.19 | 1.31 | 3.34 | 2.57 |
| BbF | 1.14 | 0.89 | 0.66 | 1.15 | 0.68 | 1.64 | 1.14 |
| BaP | 1.60 | 1.16 | 0.60 | 1.38 | 0.66 | 2.24 | 1.75 |
| BghiP | 0.05 | 0.02 | 0.04 | 0.12 | 0.12 | 0.08 | 0.05 |
| DahA | 0.19 | 0.21 | 0.17 | 0.17 | 0.16 | 0.20 | 0.03 |
| IP | 0.13 | 0.30 | 0.32 | 0.10 | 0.10 | 0.17 | 0.10 |
| Total | 15.59 | 13.04 | 10.23 | 12.14 | 8.56 | 18.17 | 10.06 |

S2C1 Table. Particle bound Polycyclic Aromatic Hydrocarbons (PPAHs) levels, in ng/m^3^, at Nursing School Makassed University (NSMU) for every sampling point (first part)

| Polycyclic Aromatic Hydrocarbons | Abbreviation | 4/12/2023 | 4/19/2023 | 5/6/2023 | 5/10/2023 | 5/11/2023 | 5/19/2023 | 5/24/2023 | 6/1/2023 |
| --- | --- | --- | --- | --- | --- | --- | --- | --- | --- |
| Naphtalene | Nap | 1.16 | 0.97 | 1.14 | 0.78 | 3.30 | 2.25 | 4.48 | 1.32 |
| Acenaphtylene | Acy | 0.52 | 0.45 | 0.51 | 0.30 | 1.12 | 0.68 | 1.87 | 0.75 |
| Acenaphtene | Ace | 0.16 | 0.14 | 0.21 | 0.17 | 0.17 | 0.27 | 0.16 | 0.15 |
| Fluorene | Flu | 0.44 | 0.42 | 0.49 | 0.41 | 0.59 | 0.45 | 0.63 | 0.48 |
| Phenanthrene | Phe | 1.47 | 0.59 | 0.94 | 0.62 | 1.72 | 0.80 | 1.40 | 0.92 |
| Anthracene | Ant | 0.53 | 0.91 | 0.77 | 0.52 | 0.97 | 0.97 | 1.01 | 0.82 |
| Fluoranthene | Flt | 1.34 | 1.83 | 1.14 | 0.44 | 1.69 | 1.22 | 2.31 | 1.27 |
| Pyrene | Pyr | 2.32 | 3.42 | 1.56 | 0.53 | 2.75 | 2.04 | 4.23 | 2.20 |
| benzo[a]anthracene | BaA | 1.97 | 1.91 | 1.11 | 0.39 | 1.79 | 1.87 | 1.72 | 1.41 |
| Chrysene | Chr | 2.97 | 3.19 | 1.80 | 0.67 | 2.49 | 2.63 | 2.72 | 1.98 |
| Benzo[k]fluoranthene | BkF | 3.69 | 5.52 | 2.23 | 14.88 | 2.79 | 2.85 | 2.75 | 1.90 |
| Benzo[b]fluoranthene | BbF | 1.89 | 2.53 | 1.00 | 7.94 | 1.36 | 1.39 | 1.32 | 0.98 |
| Benzo[a]pyrene | BaP | 2.89 | 4.42 | 1.37 | 15.26 | 2.08 | 2.31 | 2.15 | 1.50 |
| Benzo[g,h,i]perylene | BghiP | 0.13 | 1.05 | 0.77 | 3.76 | 0.07 | 0.06 | 0.12 | 0.12 |
| Dibenz[a,h]anthracene | DahA | 0.27 | 0.97 | 1.07 | 1.71 | 0.21 | 0.21 | 0.27 | 0.37 |
| Indeno[1,2,3-cd]pyrene | IP | 0.67 | 2.07 | 1.36 | 1.55 | 0.09 | 0.33 | 0.21 | 0.35 |
|  | Total | 22.42 | 30.40 | 17.48 | 49.93 | 23.21 | 20.34 | 27.35 | 16.54 |

S2C2 Table. Particle bound Polycyclic Aromatic Hydrocarbons (PPAHs) levels, in ng/m^3^, at Nursing School Makassed University (NSMU) for every sampling point (second part)

| Polycyclic Aromatic Hydrocarbons | Abbreviation | 6/7/2023 | 6/22/2023 | 6/27/2023 | 7/7/2023 | 7/12/2023 | Average in ng/m3 | Standard Deviation in ng/m3 |
| --- | --- | --- | --- | --- | --- | --- | --- | --- |
| Naphtalene | Nap | 0.98 | 0.56 | 1.51 | 1.20 | 3.88 | 1.81 | 1.27 |
| Acenaphtylene | Acy | 0.42 | 0.20 | 0.75 | 0.45 | 1.05 | 0.70 | 0.44 |
| Acenaphtene | Ace | 0.13 | 0.17 | 0.28 | 0.26 | 0.58 | 0.22 | 0.12 |
| Fluorene | Flu | 0.42 | 1.52 | 0.49 | 0.45 | 0.65 | 0.57 | 0.30 |
| Phenanthrene | Phe | 0.75 | 0.34 | 1.08 | 0.88 | 1.40 | 0.99 | 0.40 |
| Anthracene | Ant | 0.81 | 0.52 | 0.74 | 0.89 | 1.67 | 0.86 | 0.30 |
| Fluoranthene | Flt | 0.96 | 0.20 | 0.86 | 1.09 | 2.96 | 1.33 | 0.74 |
| Pyrene | Pyr | 1.45 | 0.16 | 1.85 | 1.84 | 4.95 | 2.25 | 1.34 |
| benzo[a]anthracene | BaA | 1.01 | 0.52 | 0.96 | 1.08 | 5.01 | 1.60 | 1.15 |
| Chrysene | Chr | 1.58 | 0.70 | 1.80 | 1.87 | 6.99 | 2.41 | 1.58 |
| Benzo[k]fluoranthene | BkF | 1.63 | 1.13 | 1.51 | 1.49 | 7.71 | 3.85 | 3.79 |
| Benzo[b]fluoranthene | BbF | 0.79 | 0.62 | 0.77 | 0.81 | 3.72 | 1.93 | 2.00 |
| Benzo[a]pyrene | BaP | 1.21 | 0.96 | 1.04 | 1.29 | 5.90 | 3.26 | 3.88 |
| Benzo[g,h,i]perylene | BghiP | 0.06 | 0.12 | 0.04 | 0.03 | 0.03 | 0.49 | 1.03 |
| Dibenz[a,h]anthracene | DahA | 0.20 | 0.23 | 0.17 | 0.24 | 0.31 | 0.48 | 0.47 |
| Indeno[1,2,3-cd]pyrene | IP | 0.29 | 0.25 | 0.27 | 0.08 | 0.22 | 0.59 | 0.64 |
|  | Total | 12.69 | 8.20 | 14.11 | 13.93 | 47.04 | 23.36 | 12.72 |

**S3 Text. Excess Cancer Risk (ECR)**

Equations used are equation 1 and equation 2. (the two equation are present in the manuscript)

$$\mathbf{Equation 1:} \sum BaPeq= \sum(C_{i}\times{TEF}_{i})$$

S3A Table. Application of equation 1 for the levels of PPAHs at BCD

| Polycyclic Aromatic Hydrocarbons | Abbreviation | Average in ng/m3 | TEF | Eq1 |
| --- | --- | --- | --- | --- |
| Naphtalene | Nap | 0.75 | 0.0001 | 7.46E-05 |
| Acenaphtylene | Acy | 0.38 | 0.001 | 3.78E-04 |
| Acenaphtene | Ace | 0.42 | 0.001 | 4.25E-04 |
| Fluorene | Flu | 0.60 | 0.001 | 6.01E-04 |
| Phenanthrene | Phe | 0.89 | 0.001 | 8.87E-04 |
| Anthracene | Ant | 0.76 | 0.01 | 7.63E-03 |
| Fluoranthene | Flt | 1.62 | 0.001 | 1.62E-03 |
| Pyrene | Pyr | 2.20 | 0.001 | 2.20E-03 |
| benzo[a]anthracene | BaA | 1.98 | 0.1 | 1.98E-01 |
| Chrysene | Chr | 3.16 | 0.01 | 3.16E-02 |
| Benzo[k]fluoranthene | BkF | 3.25 | 0.1 | 3.25E-01 |
| Benzo[b]fluoranthene | BbF | 1.57 | 0.1 | 1.57E-01 |
| Benzo[a]pyrene | BaP | 2.41 | 1 | 2.41E+00 |
| Benzo[g,h,i]perylene | BghiP | 0.13 | 0.01 | 1.33E-03 |
| Dibenz[a,h]anthracene | DahA | 0.24 | 1 | 2.35E-01 |
| Indeno[1,2,3-cd]pyrene | IP | 0.16 | 0.1 | 1.60E-02 |
|  | Total |  |  | 3.39E+00 |

$$\mathbf{Equation 2:} Excess Cancer Risk \left( ECR \right)= \sum BaPeq \times{UR}_{BaP}$$

Using equation 2:

Excess cancer risk at BCD= 3.39* 0.0000011= **3.73x10^-6^**

S3B Table. Application of equation 1 for the levels of PPAHs at AUB

| Polycyclic Aromatic Hydrocarbons | Abbreviation | Average in ng/m3 | TEF | Eq1 |
| --- | --- | --- | --- | --- |
| Naphtalene | Nap | 0.75 | 0.0001 | 7.45E-05 |
| Acenaphtylene | Acy | 0.38 | 0.001 | 3.68E-04 |
| Acenaphtene | Ace | 0.42 | 0.001 | 5.53E-04 |
| Fluorene | Flu | 0.60 | 0.001 | 4.29E-04 |
| Phenanthrene | Phe | 0.89 | 0.001 | 7.30E-04 |
| Anthracene | Ant | 0.76 | 0.01 | 7.83E-03 |
| Fluoranthene | Flt | 1.62 | 0.001 | 1.32E-03 |
| Pyrene | Pyr | 2.20 | 0.001 | 1.71E-03 |
| benzo[a]anthracene | BaA | 1.98 | 0.1 | 1.35E-01 |
| Chrysene | Chr | 3.16 | 0.01 | 2.50E-02 |
| Benzo[k]fluoranthene | BkF | 3.25 | 0.1 | 3.34E-01 |
| Benzo[b]fluoranthene | BbF | 1.57 | 0.1 | 1.64E-01 |
| Benzo[a]pyrene | BaP | 2.41 | 1 | 2.24E+00 |
| Benzo[g,h,i]perylene | BghiP | 0.13 | 0.01 | 8.10E-04 |
| Dibenz[a,h]anthracene | DahA | 0.24 | 1 | 2.03E-01 |
| Indeno[1,2,3-cd]pyrene | IP | 0.16 | 0.1 | 1.72E-02 |
|  | Total |  |  | 3.13E+00 |

Using equation 2:

Excess cancer risk at AUB= 3.13* 0.0000011= **3.44x10^-6^**

S3C Table. Application of equation 1 for the levels of PPAHs at NSMU

| Polycyclic Aromatic Hydrocarbons | Abbreviation | Average in ng/m3 | TEF | Eq1 |
| --- | --- | --- | --- | --- |
| Naphtalene | Nap | 1.81 | 0.0001 | 1.81E-04 |
| Acenaphtylene | Acy | 0.70 | 0.001 | 6.98E-04 |
| Acenaphtene | Ace | 0.22 | 0.001 | 2.20E-04 |
| Fluorene | Flu | 0.57 | 0.001 | 5.74E-04 |
| Phenanthrene | Phe | 0.99 | 0.001 | 9.94E-04 |
| Anthracene | Ant | 0.86 | 0.01 | 8.57E-03 |
| Fluoranthene | Flt | 1.33 | 0.001 | 1.33E-03 |
| Pyrene | Pyr | 2.25 | 0.001 | 2.25E-03 |
| benzo[a]anthracene | BaA | 1.60 | 0.1 | 1.60E-01 |
| Chrysene | Chr | 2.41 | 0.01 | 2.41E-02 |
| Benzo[k]fluoranthene | BkF | 3.85 | 0.1 | 3.85E-01 |
| Benzo[b]fluoranthene | BbF | 1.93 | 0.1 | 1.93E-01 |
| Benzo[a]pyrene | BaP | 3.26 | 1 | 3.26E+00 |
| Benzo[g,h,i]perylene | BghiP | 0.49 | 0.01 | 4.89E-03 |
| Dibenz[a,h]anthracene | DahA | 0.48 | 1 | 4.78E-01 |
| Indeno[1,2,3-cd]pyrene | IP | 0.59 | 0.1 | 5.94E-02 |
|  | Total |  |  | 4.58E+00 |

Using equation 2:

Excess cancer risk at NSMU= 4.58* 0.0000011= **5.04x10^-6^**

**S4 Text. Contribution of individual PPAHs on the excess cancer risk**

Benzo[a]pyrene, Benzo[k]fluoranthene, Dibenz[a,h]anthracene, Benzo[b]fluoranthene, and Benzo[a]anthracene had a great contribution on the excess cancer.

S4 Table. Calculation of percent contribution of every individual PPAH in the excess cancer risk calculated.

| **Species** | **Escess Cancer Risk** | **Location** |  | **Percent Contribution** |
| --- | --- | --- | --- | --- |
| Others | 6.97E-08 | BCD |  | 2 |
| Benzo[a]anthracene | 2.18E-07 | BCD |  | 6 |
| Benzo[k]fluoranthene | 3.58E-07 | BCD |  | 10 |
| Benzo[b]fluoranthene | 1.73E-07 | BCD |  | 5 |
| Benzo[a]pyrene | 2.65E-06 | BCD |  | 71 |
| Dibenz[a,h]anthracene | 2.64E-07 | BCD | 3.73E-06 | 7 |
| Others | 6.21E-08 | AUB |  | 2 |
| Benzo[a]anthracene | 1.49E-07 | AUB |  | 4 |
| Benzo[k]fluoranthene | 3.67E-07 | AUB |  | 11 |
| Benzo[b]fluoranthene | 1.80E-07 | AUB |  | 5 |
| Benzo[a]pyrene | 2.46E-06 | AUB |  | 72 |
| Dibenz[a,h]anthracene | 2.20E-07 | AUB | 3.44E-06 | 6 |
| Others | 1.15E-07 | NSMU |  | 2 |
| Benzo[a]anthracene | 1.76E-07 | NSMU |  | 3 |
| Benzo[k]fluoranthene | 4.24E-07 | NSMU |  | 8 |
| Benzo[b]fluoranthene | 2.12E-07 | NSMU |  | 4 |
| Benzo[a]pyrene | 3.59E-06 | NSMU |  | 71 |
| Dibenz[a,h]anthracene | 5.28E-07 | NSMU | 5.04E-06 | 10 |

Others are the following: Indeno[1,2,3-cd]pyrene, Chrysene, Benzo[g,h,i]perylene, Pyrene, Fluoranthene, Naphtalene, Phenanthrene, Fluorene, Acenaphtylene, Acenaphtene, and Anthracene.

Percent Contribution is calculated by dividing the excess cancer of an individual PPAH by the total excess cancer risk then multiplied by 100.
