# Supplementary material for: When do science recommendations stop being effective? The case of the sprawl of diesel electricity generators in Beirut
Source: PLoS One. 2024 Dec 31;19(12):e0313341. doi: 10.1371/journal.pone.0313341 (PMC11687642; doi:10.1371/journal.pone.0313341)
Supplement: Attachment — Submitted filename: Response to Reviewers.docx [file pone.0313341.s002.docx]

Reviewer 1:

Thank you for your insightful feedback and constructive comments on my manuscript. Your suggestions will undoubtedly enhance the quality of the work.

Comments:

This manuscript presents a source apportionment study on a considerably long dataset at multiple sites in Beirut using polycyclic aromatic hydrocarbons as tracers. The study was performed by using positive matrix factorization applied to original data to evaluate the contribution of diesel electricity generators to polycyclic aromatic hydrocarbons. Furthermore, the excess cancer risk is calculated. The results provide interesting information about the sources of polycyclic aromatic hydrocarbons in Beirut. The manuscript is overall written in clear English, although the “Methods” and “Results” could benefit from a revision especially focused on the findings represented (see my specific comments below). I believe this manuscript is of interest to the scientific community and is coherent with the aims of this journal. Nevertheless, in my opinion, a few issues need to be addressed before the manuscript can be accepted for publication.

1- Lines 37 Please define the name of the sampling site when they are shown first.

We thank the reviewer for this comment. This issue is fixed in the revised text, as follow:

” . The locations are: American University of Beirut (AUB), Beirut Central District (BCD) and Nursing School Makassed University (NSMU).”

2- Lines 183-189 The descriptions of source apportionment should be shown in detail. The current version is too simple to follow for the readers.

We thank the reviewer for this comment. The descriptions were added in the revised text, as follow:

“. It explains the data set variability with a linear combination of factor profiles (sources) ${(f}_{i,k})$ representing the chemical tracers (PAHs) and their varying contribution ($g_{k,j}$). The resultant residual matrix is termed as ($e_{i,j}$). $The input matrix is represented by {(x}_{i,j}).$The indices $i \mathrm{and} j$ $\mathrm{represent}$the number of samples and the number of chemical species respectively. The number of sources is represented by ($p)$ $and is determined and adjusted manually depending on the$ expectation from the sampling sites and their realistic environmental interpretation.

In this case, the equation below is explained by the input matrix being our PPAHs complete data set (from the three sites), and the result is the factor profiles ($f_{i,k}$), the time series ($g_{k,j}$), and a residual matrix ($e_{i,j}$).

$$x_{i,j}= \sum_{k=1}^{p} \left( f_{i,k}\cdot g_{k,j} \right)+e_{i,j}$$

”

3- The descriptions of cancer risk calculation should be shown in detail. The current version is too simple for the readers to follow.

We thank the reviewer for this comment. The descriptions were added in the revised text, as follow:

“

$$\mathbf{Equation 1:} \sum BaPeq= \sum(C_{i}\times{TEF}_{i})$$

Where $\sum BaPeq$ is the total BaP equivalent concentrations, C_i_ is the concentration of each PAH in ng/m^3^, and TEFi is the toxicity equivalent factor of each PAH. (28-30)

To evaluate the potential risk of developing cancer through inhalation and exposure to particle-bound PAHs, the OEHHA procedure was utilized. This established approach allows for the calculation of the lifetime excess cancer risk using the following equation:

$$\mathbf{Equation 2:} Excess Cancer Risk \left( ECR \right)= \sum BaPeq \times{UR}_{BaP}$$

The equation takes into consideration the UR_BaP_ value, which represents the number of individuals at risk of developing cancer from inhaling a BaP equivalent concentration of 1 ng/m^3^ over a 70-year lifetime.(31, 32) The value of UR_BaP_ utilized in this study was $1.1\times{10}^{-6}$ (equivalent to 0.11 cases per 100,000 people), as determined by the OEHHA of the CalEPA.”

4- Lines 199. There is no figure 2.

We thank the reviewer for this comment. The figure was re-uploaded

5- Line 268. The quality of Figure 4 should be improved. The authors should present the source profiles for each factor.

We thank the reviewer for this comment. The figure was re-uploaded but it seems the quality will not be improved since it is an issue in uploading. The figure will be re-uploaded with full quality and maximum possible resolution.

6- Lines 273-286. There are no discussions for the source profile of each profile at all. The authors should compare the source profiles in this study with prior results in other studies

Thank you for your comment. Upon reviewer suggestion, references were splitted.

7- Lines 342-351. What are the energy policies recommended from the authors? Biomass has been regarded as a promising option for solid energy, which is promoted in the residential sector and firing power plants in some low-income and middle-income counties. The established programs and technologies of wood control burning requirements have been documented in the US, which contribute to the major efforts in regulating air quality in the Northeastern US where wood fuel is very important

We thank the reviewer for this comment. Lebanon is living an economic crisis which led to blackouts in the country. Diesel generators are forming an independent and uncontrolled private sector. The recommendation first as mentioned in the last paragraph that electricity is a right and should be provided by the national provider and not a private sector with uncontrolled prices. People, who can afford, depend on solar panels installation. As a conclusion, the electricity situation in Lebanon is worse to the level where the Lebanese population hope that the national provider use his power plant generators, who work on imported heavy fuel oil, just to get rid of unsafe spread of diesel generators between buildings and on their rooftops.

Lebanon is facing a severe economic crisis that has led to widespread blackouts across the country. In response, diesel generators have formed an uncontrolled private sector, with citizens forced to pay exorbitant prices for basic electricity. Those who can afford it have resorted to solar panel installations as a temporary solution, but this does not address the fundamental issue: electricity is a right that must be provided by the government.

The parliament has taken some steps forward by approving a renewable energy law that regulates the integration of renewable sources as an alternative to the failing national grid. This law also establishes guidelines for public-private partnerships to facilitate the installation of small- and medium-scale power plants. In parallel, efforts are being made to rehabilitate several hydropower stations.

The widespread reliance on diesel generators across rooftops and between buildings has placed a significant strain on public health and well-being. The constant hum of these generators has turned the city into a chaotic environment that is becoming unbearable. A solution must be urgently implemented—a reliable, government-controlled electricity supply is essential to restore order and improve the quality of life for all.

Reviewer 2:

Your thorough review and valuable insights on the manuscript are sincerely appreciated. Your feedback will be instrumental in improving the overall clarity and impact of the paper.

Comments:

The studies tackles an extremely important environmental issue, linking economic recession and fuel poverty with carcinogenic aerosol pollution, in a semi-quantitative manner. However, the approach taken is rather local, and the discussion is very limited in terms of comparisons with the international status. The source apportionment methodology should be explained in more detail, and the scope of PMF related analysis and valorization of data should be expanded. The manuscript must be revised before publication according to the specific comments listed below.

1- L58-60: Since the national provider was totally shut down, how is power provided during non-blackout periods? Are there secondary providers?

Thank you for your comment. Électricité du Liban (EDL), the national provider, has struggled to provide consistent electricity, forcing both households and public sector institutions to rely on diesel generators to keep functioning. During total blackouts, people are left with two difficult choices: either live in darkness for few hours or depend on a few solar panels installed on their balconies, if they are fortunate enough to have the space. The lack of a reliable national power supply has driven citizens to adopt these makeshift solutions, highlighting the urgent need for a sustainable resolution to Lebanon's worsening energy crisis.

This text was added to the introduction (Lines 127-131).

2- L66-67: What about the remaining 63%?

Thank you for your comment. By 2019, EDL’s power plants produce around 63% of the country’s total electricity demand whereas diesel generators contribute 8.1 terawatt-hours (~37 percent) according to a recent World Bank study.

This text was added to the introduction (Lines 75-79).

3- Introduction: The study highlights a very important subject, but the presentation is local in scope. The introduction should broaden the implications of this type of research by presenting similar impacts of fuel poverty in other areas of the world. Such an example is the severe air quality degradation in European cities due to the EU debt crisis that led to the increase of oil prices and the widespread use of biomass products for heating (see, for example, Kaskaoutis et al., 2021, Atmos. Environ.). Another is the use of extremely polluting, low-quality farming byproducts for cooking and heating purposes in low-income countries that rely on an agrarian economy (Saenz et al., 2021, Environ. Int.; Bhattu et al., 2024, Nat. Comm.). Include a brief introductory paragraph in this direction to place the study within an international context.

Thank you for your comment. Two paragraphs were added to the Introduction, as follow:

“PPAHs are an environmental concern specifically in countries dealing with economic crises. PPAHs can have detrimental effects on the environment and human health. The economic crises can impact energy policies, leading to increased reliance on cheaper but environmentally harmful energy sources like diesel generators, wood and biomass burnings and waste incinerations.

4- L122: Indicate the distance of the highway and whether it affects measurements.

Thank you for your comment. The distance was accounted for 130m and it was added to the revised text. Regarding its impact on the measurements, a recent study by Jaafar et al. (2018) identified gasoline as a significant source of PAH concentrations in the air at this site, with a source contribution of 48%.

5- L122-126: So, what is the type of this site? Still urban background but more central or traffic?

Thank you for your comment. The second site in BCD is still considered an urban background location. It is situated in a pedestrian-only area and the nearest highway is approximately 250 meters away from the site. This text was added. (Lines 152-154)

6- L129: Indicate the distance and traffic load of this road and whether they result in a “traffic” site characterization.

Thank you for your comment. This location is influenced by emissions from diesel generators and vehicular traffic due to its proximity by 10 meters to the principal roadway with heavy car traffic. This text was added (Lines 156-157)

7- L131: It is unclear how this sampling strategy will help assess the generators' impact. This is the main feature of the paper and must be explained. In addition, the sample numbers per site/period must be provided

Thank you for your comment. Given the widespread presence of diesel generators across most city neighborhoods, the sampling strategy described in the manuscript was designed to capture data from three distinct types of neighborhoods: 1) a high socio-economic status area and business district with a demand for large, high-powered generators, 2) a densely populated, lower socio-economic status area with heavy traffic, and 3) a university site in the city surrounded by greenery. Sampling was conducted at these three locations throughout an entire year to account for temporal variations in pollutant levels at specific sites where generators are frequently in use. By collecting 24-hour samples once every six days, we aimed to create a comprehensive dataset that reflects the fluctuations in pollutant emissions from generators over time.

This text was added. (Lines 158-167)

The number of samples per site was also included to ensure robust data collection.(Lines 283, 286 and 289)

8- L139: Did you use denuders or impregnated backup filters in the Chemcomb?

Thank you for your comment. Teflon PTFE (polytetrafluoroethylene) membrane filters (TISCH Scientific) were used in order to collect air then weigh the filter in order to identify the concentration. The cartridge was used in lieu of the PM impactor with plastic rings and meshes used to hold the filter and no denuders were used to capture the gases.

9- L176-177: It would be more meaningful if you expressed these values per m3 of sampled air (actual or nominal).

Thank you for your comment. PM_2.5_ concentrations were reported in m^3^ of sampled air of course. The extracted solutions were expressed in ml of solution but then once the PPAHs are quantified they are converted back to m^3^ per air.

10- L182: So, did you perform any blank corrections?

Thank you for your comment. All quality control and quality assurance procedures were completed and assessed multiple times during the sampling and analysis period. Analysis of the blank filters was a routine procedure we undertook and corrections when needed were taken. In all cases the blank filter did not show any traces of the Blank investigation was done. However, the 16 PPAHs were not found in the blank filters. A sentence in this regard was added to the text (Lines 221-222)

11- L173-182: Was it possible to calculate the repeatability of your quantifications using replicate analyses? That would be useful for the PMF as well.

Thank you for your comment. The repeatability is a routine procedure we do with the GC-MS analysis. It is reflected in the reported %RSD which is usually assessed by repeating the whole extraction and analysis procedure. The sentence present in the text reflects this step: The results of seven replicate standards for two concentrations (0.1 and 4 μg/mL) revealed % RSD (percent relative standard deviation) less than 7% shows that seven replicate standards were tested at two different concentrations and the repeatability was less than 7%.

12- L183-189: The methodological presentation of the source apportionment is insufficient. All necessary details must be provided, including uncertainty calculations, model selection strategy, model evaluation, error evaluation, rotations, constraints etc. This section must be substantially expanded. It is also important to indicate if the PAH dataset from three sites was combined to perform the PMF analysis.

Thank you for your comment. The methodological part was expanded and the last sentence, mentioned that the complete data set was used, was added to in the revised text as follow:

” . It explains the data set variability with a linear combination of factor profiles (sources) ${(f}_{i,k})$ representing the chemical tracers (PAHs) and their varying contribution ($g_{k,j}$). The resultant residual matrix is termed as ($e_{i,j}$). $The input matrix is represented by {(x}_{i,j}).$The indices $i \mathrm{and} j$ $\mathrm{represent}$the number of samples and the number of chemical species respectively. The number of sources is represented by ($p)$ $and is determined and adjusted manually depending on the$ expectation from the sampling sites and their realistic environmental interpretation.

In this case, the equation below is explained by the input matrix being our PPAHs complete data set (from the three sites), and the result is the factor profiles ($f_{i,k}$), the time series ($g_{k,j}$), and a residual matrix ($e_{i,j}$).

$$x_{i,j}= \sum_{k=1}^{p} \left( f_{i,k}\cdot g_{k,j} \right)+e_{i,j}$$

”.

13- L195-196: Not enough. You should list the TEFs used and their bibliographical sources. Also, indicate the used CalEPA unit risk. You should also mention that BaPeq is calculated as an intermediate step.

Thank you for your comment. The cancer risk part was expanded to represent the calculations steps as follow:”

$$\mathbf{Equation 1:} \sum BaPeq= \sum(C_{i}\times{TEF}_{i})$$

Where $\sum BaPeq$ is the total BaP equivalent concentrations, C_i_ is the concentration of each PAH in ng/m^3^, and TEFi is the toxicity equivalent factor of each PAH. (28-30)

To evaluate the potential risk of developing cancer through inhalation and exposure to particle-bound PAHs, the OEHHA procedure was utilized. This established approach allows for the calculation of the lifetime excess cancer risk using the following equation:

$$\mathbf{Equation 2:} Excess Cancer Risk \left( ECR \right)= \sum BaPeq \times{UR}_{BaP}$$

The equation takes into consideration the UR_BaP_ value, which represents the number of individuals at risk of developing cancer from inhaling a BaP equivalent concentration of 1 ng/m^3^ over a 70-year lifetime.(31, 32) The value of UR_BaP_ utilized in this study was $1.1\times{10}^{-6}$ (equivalent to 0.11 cases per 100,000 people), as determined by the OEHHA of the CalEPA.”.

Additionally, 3 references were added as references that include the list and numerical value of TEF for every individual PAH.

14- L198: This section should discuss the factors i.e. meteorological, varying seasonal emissions, spatial source constrasts, to explain the differences observed between the three sites-periods. This is necessary because if the site characteristics are widely different there is no point in calculating annual mean PM2.5 or using a combined dataset in the PMF.

We totally agree with the reviewer’s comments and this comment might have been raised because Figure 2 was not uploaded. We apologize for this inconvenience and hope Figure 2 uploaded this time will clearly show the difference in the sites. In addition to Figure 2 we have included in the first paragraph of the result section the averages in each site and explained how they differ from each other. Also in the PMF results, the analysis was done for each site alone as indicated by the reviewer.

One thing we did after we have shown the particularity of each site is we combined the data to report the overall average of the city of Beirut.

15- L217-218: What about the other two?

Thank you for your comment. The percentages of the other two sites (AUB and BCD) were added and the paragraph was paraphrased in order to be clearer.

16- L235-236: However, there is substantial site variation per member. This should be discussed. Moreover, the heavy members appear to record the higher levels at MSNU. It should be hinted early why this is the case (traffic or diesel generators?).

Thank you for your comment. As recommended, members of PPAH of toxicity higher or equal to 0.1 according to the TEF chart are compared among the three sites and added to the PPAH result section as follows:

Benzo[a]Pyrene (TEF = 1) concentrations increased as we moved from a site with greenery, but influenced by traffic and a nearby generator (AUB, 2.24 ng/m^3^), to a business site with heavy diesel generator use and minimal traffic since it's a pedestrian-only area (BCD, 2.41 ng/m^3^), and then to a site with significant diesel generator use and high traffic (NSMU, 3.26 ng/m^3^). The trend also showed higher levels of PPAHs at NSMU compared to AUB and BCD. For example, Benzo[k]fluoranthene (TEF = 0.1) was measured at 3.34 ng/m^3^ at AUB, 3.25 ng/m^3^ at BCD, and 3.85 ng/m3 at NSMU. Benzo[b]fluoranthene (TEF = 0.1) concentrations were 1.64 ng/m^3^ at AUB, 1.57 ng/m^3^ at BCD, and 1.93 ng/m^3^ at NSMU. Similarly, Indeno[1,2,3-cd]pyrene (TEF = 0.1) were recorded at 0.17 ng/m^3^ at AUB, 0.16 ng/m^3^ at BCD, and 0.64 ng/m^3^ at NSMU.

17- L241-242: Specify that this is a “cooling” demand

Thank you for your comment. It was added to the revised text as follow:” This heightened demand is particularly pronounced during the hot summer season for cooling purposes.”.

18- L247-249: What type of hypothesis testing is used? This should be relevant also to the sample sizes.

Thank you for your comment. It was added to the revised text, as follow:” After conducting T.test, the data revealed a significant difference between the air quality at AUB and BCD areas (p = 0.01), as well as between AUB and NSMU (p = 0.001), with a lesser disparity observed between BCD and NSMU (p = 0.07)”.

19- Table 1: Check “NSMU”.

Done.

20- L254-257: Indicate which is the relative effect of traffic at BCD and MSNU.

Thank you for your comment. It was added to the revised text as follow:” Conversely, BCD areas typically host a higher number of diesel generators with greater capacities operating for longer durations and surrounded by highways with moderate to low load of traffic. Similarly, NSMU exhibits fewer green spaces and a higher concentration of diesel generators relative to AUB and a higher load of traffic, which contributes to its distinct air quality profile.”.

21- L262: The way the references are used in this section is problematic. You should indicate specific references for each of the three factors and discuss the ones supporting your characterization. Focus especially on PAH PMF studies in the Eastern Mediterranean and the Middle East. This is important for all three factors.

Thank you for your comment. Upon reviewer suggestion, references were splitted.

22- L275-277: There has been no indication up to now for this type of source affecting the data in the text, and it is unknown how it can impact the area and under which conditions. In general, you should link the factor contributions with wind data (e.g. are the sites downwind of the incinerators when high factor contributions are estimated?) and also consider the seasonal (spatial) variability of source contributions. The discussion at present does not support the factor characterization enough. For example, based on this profile, Factor 2 could also be related to biomass burning emissions.

Thank you for your comment. Factor 1 predominantly featured medium molecular weight compounds (MMW) such as Fluoranthene, Pyrene, Benzo[a]anthracene, and Chrysene, alongside Benzo[k]fluoranthene, indicative of emissions from diesel engines and generators. Factor 2 was characterized by high levels of Benzo[k]fluoranthene, Benzo[b]fluoranthene and, Benzo[a]pyrene, Benzo[g,h,i]perylene, Dibenz[a,h]anthracene, and Indeno[1,2,3-cd]pyrene, consistent with incinerator emissions, particularly medical ones. Factor 3 exhibited high loadings of Dibenz[a,h]anthracene and Indeno[1,2,3-cd]pyrene, with moderate loading of benzo[ghi]perylene, alongside elevated levels of low molecular weight PPAHs, notably naphthalene, indicative of gasoline emissions.

To confirm the assignment of Factor 1 to diesel emissions, the lower content of Ind and BghiP is used as it represents one of the main differences in PAHs content between gasoline exhaust and diesel exhaust since gasoline exhaust is formed by pyrosynthesis as opposed to diesel exhaust where the unburnt fuel content in PAHs is much more important. Also, to differentiate between diesel (Factor 1) and gasoline emission (Factor 3), some studies use diagnostic ratios to confirm their identifications. As example, the ratio between BaP and Chr (BaP/(BaP + Chr) = 0.4) is lower than 0.5 which support the association of Factor 1 with diesel emissions (#Cerqueira et Matos, 2019). Additionally, (BaA/BaA+BaP) ratio was around 0.43 which indicates diesel burnings contribution (R. Baalbaki et al. 2018 and Q. Bian et al. 2016).

In addition, the concentrations of Flt and Pyr are the highest among the three sources, also factor 3 has a substantial loading in Baa, Chr, BkF, BbF and some BaP compared to gasoline vehicles factor where the concentrations of Ind and BghiP contribute the most (Manoli et al 2002, Iakovides 2019) While acknowledging the reviewer's concern that factor 2 could potentially be associated with biomass burnings, our analysis suggests a stronger correlation with incinerators. Upon closer examination of the emission sources linked to factor 2, the data indicated possible connections to both incinerators and biomass burning. However, a focused study of the environmental context in Beirut and its neighboring cities led us to dismiss biomass burnings as the primary source. The absence of a consistent pattern or significant instances of biomass burning in the region further supports our assertion that factor 2 is more likely attributed to incinerator emissions.

23- L277-286: In general, gasoline cars tend to produce more heavy than light PAHs. Are there any diesel-powered trucks and other HDDVs in Beirut? It could be that this is a combined diesel-gasoline traffic factor. I would not consider naphthalene as a tracer, as its overwhelming majority is in the gas phase, and its particle fraction is extremely unstable; therefore, not suitable for PMF (same goes for Ace, Acy and probably Flu, Phe). Check also the seasonality of the factor.

Thank you for your comment. Based on the reviewer comments, we reformulated the PMF analysis using PAHs other than Naphthalene to support the factor assignments and the interpretation. Kindly see the answer shown in comment 20 above. Additionally, to confirm the contribution of diesel burnings in emitting heavy PAHs, a ratio of (IP/IP+BghiP) valued between 0.5 and 0.7 which indicates the diesel contribution. Moreover, (BaA/BaA+BaP) ratio was around 0.43 which indicate diesel burnings contribution (R. Baalbaki et al. 2018). Additionally, a sentence was added that includes that it is quite unusual to see diesel trucks making their way near the three specific sites.

24- L288-292: The source contributions should be calculated per site, and the spatial contrasts should be discussed. This also helps verify the sources. Consider this in the discussion.

Thank you for your comment. The source contribution was done for each site as shown in Figure 5. As suggested by the reviewer, the comparison between the different sites and the comparison between the study done for this year and previous years was added as shown below:(Line 400-406)

Furthermore, there has been a notable decline in the proportion of emissions attributed to gasoline and incineration, dropping from 48% to 36% and 29% to 21%, respectively. This shift can be primarily attributed to the significant increase in operational hours of diesel generators, rising from 3 hours to 18 hours per day. Consequently, diesel combustion emissions have emerged as a more substantial contributor to pollution levels when compared to the findings of the earlier study conducted in 2017-2018.

25- L293: Why didn’t you calculate BaPeq and risks for each of the three sources? Using the source profiles, this is feasible. Consider this also in the discussion.

Thank you for your comment and suggestion. Your suggestion is answered in Figure 6 where the contribution of the PPAHs with higher toxicity were shown for every sources.

26- L314-324: These have already been discussed; take a more general approach.

Thank you for your comment. Done in the revised text as follow:” The evaluation of PPAHs concentrations at three separate locations in Beirut indicated notable variations over time and between sites, potentially posing carcinogenic risks. These levels tended to be higher during periods of lower temperatures, aligning with previous research findings. (13, 15, 16, 48)”.

27- L326-327: Indicate that you are comparing to a study sampling PM10, not PM2.5.

Thank you for your comment. It is true that should be included. However, in general PAHs tend to be in particulate matter of size 2.5 micrometer or less.(N. Daher et al. 2013 and baal).

28- L342-346: Political statements that are not supported by references. You have to rephrase them in scientific language.

Thank you for your comment. Done in the revised text as follow:” Despite repeated recommendations and academic presentations, the local government has chosen to ignore these warnings. This has resulted in a sector of electricity producers and merchants operating outside the law, with no oversight from any government entity.”.

29- L346-347: Add reference.

Thank you for your comment. Done in the revised text.

30- L354-357: Very important conclusion, include it in the abstract.

Thank you for your comment. Done in the revised text as follow:” This situation, where law of enforcement is absent, need for international action to curb emissions and for funding agencies to adopt sustainable, "carbon-free" funding strategies to support urban development in low- and middle-income countries (LMICs). Yet, EDL's failure to fulfill Lebanon's populace electricity requirements infringes upon their electricity entitlements.”.
